# Beyond the Usual Suspects: Unlocalizable Back Pain After Trauma

**DOI:** 10.1016/j.acepjo.2025.100292

**Published:** 2025-12-20

**Authors:** Julia Isaacson, Jennifer Frush, Andrew Mittelman

**Affiliations:** Boston Medical Center, Department of Emergency Medicine, Boston, Massachusetts, USA

**Keywords:** trauma, back pain, aortic dissection

## 1 Case Presentation

**Figure 1:**
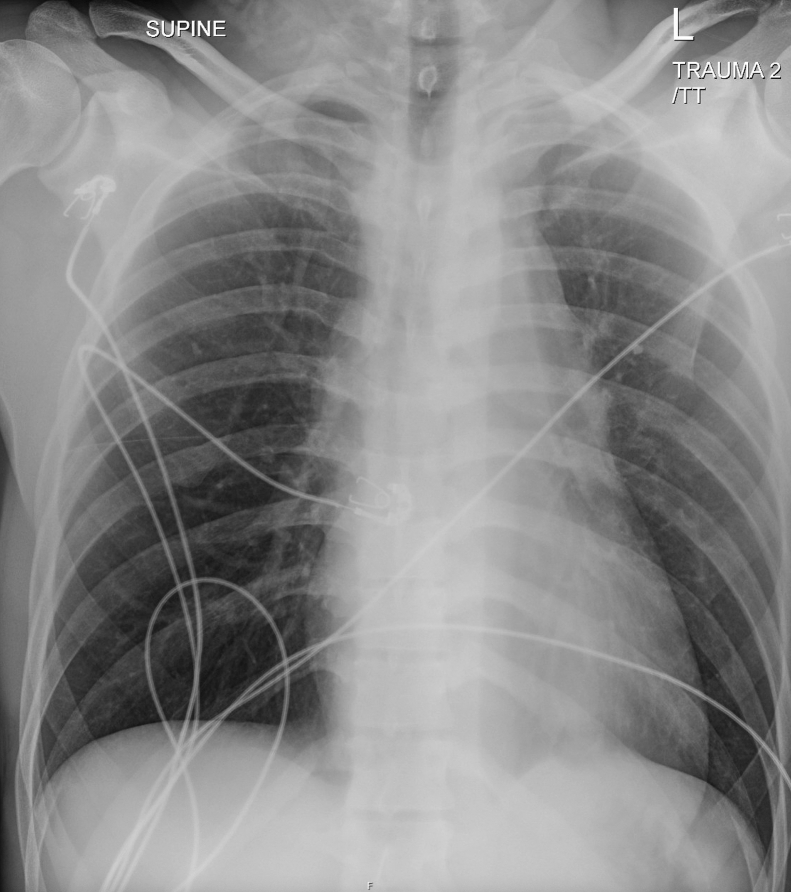
Chest radiograph demonstrating mediastinal widening and loss of the aortic knob.

**Figure 2:**
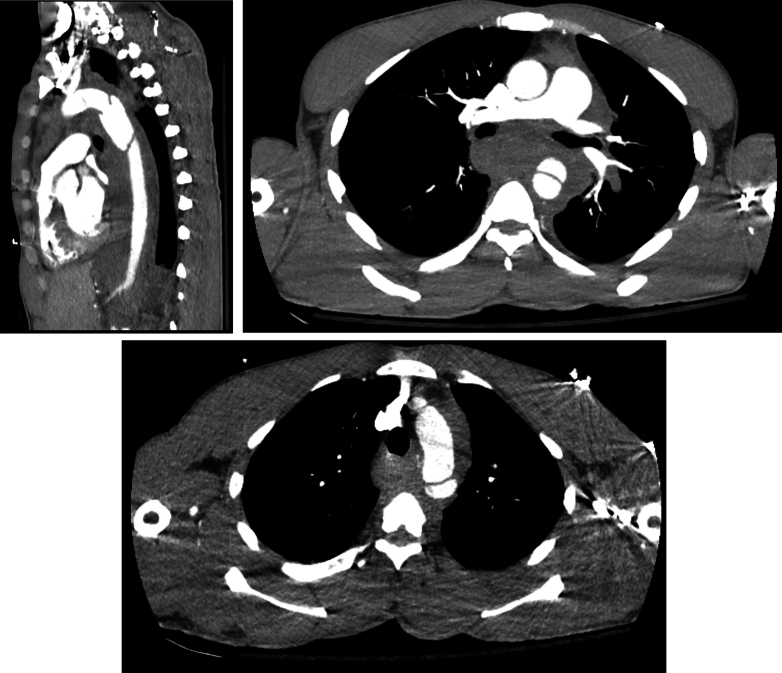
Contrast-enhanced CT of chest, abdomen, and pelvis revealing descending thoracic aortic pseudoaneurysm with an intimal flap, extensive hemomediastinum, and anterior heart displacement.

A previously healthy 38-year-old man presented with severe back pain after jumping from a third-story window. He reported a head strike but denied loss of consciousness and was ambulatory at the scene. On arrival, he was alert but uncomfortable. His airway was intact, lungs were clear, and he was hemodynamically stable. A secondary survey revealed minor abrasions and lacerations. No midline spinal tenderness or step offs were noted. Peripheral pulses were intact, and there were no motor or sensory deficits. His eFAST was negative. A portable chest x-ray (Fig 1) and a computed tomography (CT) of the chest, abdomen, and pelvis (Fig 2) were obtained.

## 2 Diagnosis

### 2.1 Traumatic Aortic Dissection

An esmolol infusion was started in the emergency department, and the patient underwent emergent thoracic endovascular aortic repair. He was discharged to rehabilitation one week later without any major complications.

### 2.2 Teaching Points

Traumatic aortic injury is rare, occurring in <1% of blunt trauma cases, but is highly lethal, with up to 85% of patients dying at the scene.^1^ It is the second leading cause of death in blunt trauma behind traumatic brain injury. CT angiography is the diagnostic modality of choice, with sensitivity and specificity approaching 100%.^2^ In contrast, chest radiography—used to assess for indirect signs of aortic injury such as mediastinal widening—has low sensitivity (∼40%). Traumatic aortic injury grades II to IV generally require operative repair, and endovascular approaches are typically preferred.^3^

## Funding and Support

By *JACEP Open* policy, all authors are required to disclose any and all commercial, financial, and other relationships in any way related to the subject of this article as per ICMJE conflict of interest guidelines (see www.icmje.org). The authors have stated that no such relationships exist.
